# Decompensation of ****β****-Cells in Diabetes: When Pancreatic ****β****-Cells Are on ICE(R)

**DOI:** 10.1155/2014/768024

**Published:** 2014-02-10

**Authors:** Roberto Salvi, Amar Abderrahmani

**Affiliations:** ^1^European Genomic Institute for Diabetes (EGID), Lille 2 University, UMR 8199, 3508 Lille, France; ^2^Faculty of Medicine West, 1 Place de Verdun, 59045 Lille, France

## Abstract

Insulin production and secretion are temporally regulated. Keeping insulin secretion at rest after a rise of glucose prevents exhaustion and ultimately failure of **β**-cells. Among the mechanisms that reduce **β**-cell activity is the inducible cAMP early repressor (ICER). ICER is an immediate early gene, which is rapidly induced by the cyclic AMP (cAMP) signaling cascade. The seminal function of ICER is to negatively regulate the production and secretion of insulin by repressing the genes expression. This is part of adaptive response required for proper **β**-cells function in response to environmental factors. Inappropriate induction of ICER accounts for pancreatic **β**-cells dysfunction and ultimately death elicited by chronic hyperglycemia, fatty acids, and oxidized LDL. This review underlines the importance of balancing the negative regulation achieved by ICER for preserving **β**-cell function and survival in diabetes.

## 1. Introduction

The exposure of population to overfeeding and sedentary lifestyles has increased dramatically during the last decades worldwide. This has been accompanied by a rise in the incidence of obesity and therefore the associated morbidity and mortality. These complications are related to comorbid conditions including diabetes. Insulin resistance is the most common metabolic alteration related to obesity and is considered to be a critical link between adiposity and the risk for developing diabetes. However, in most of cases, obesity does not lead to diabetes. This situation is thought to result from the capacity of *β*-cells to compensate for insulin resistance by releasing appropriate amount of insulin in blood probably by an increased *β*-cells function and mass. When the cells decompensate and thereby fail to secrete adequate insulin in the face of increased hormone demand, then there, overt diabetes comes. In this respect, identification of leading mechanisms that account for *β*-cells compensation and decompensation would permit to pave the way for innovative therapeutic strategies of diabetes. The present review unveils a role for the cAMP pathway target inducible cAMP early repressor (ICER) as a central player for *β*-cells adaptation, which is impinged in *β*-cells under diabetes environmental stressors.

## 2. Portrait of ICER

**Figure 1:**
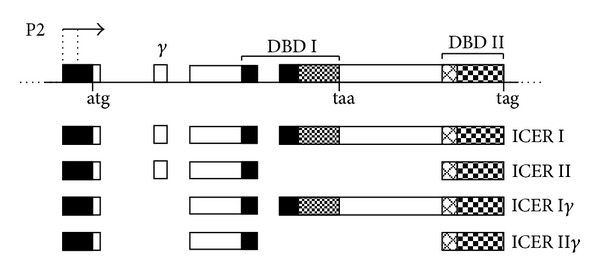
Expression of the ICER isoforms. ICER results from the P2 alternative promoter within the CREM gene. The ICER I and II isoforms are the results of alternative splicing. ICER I*γ* and II*γ* have the *γ* exon. DBD: DNA binding domains.

**Figure 2:**
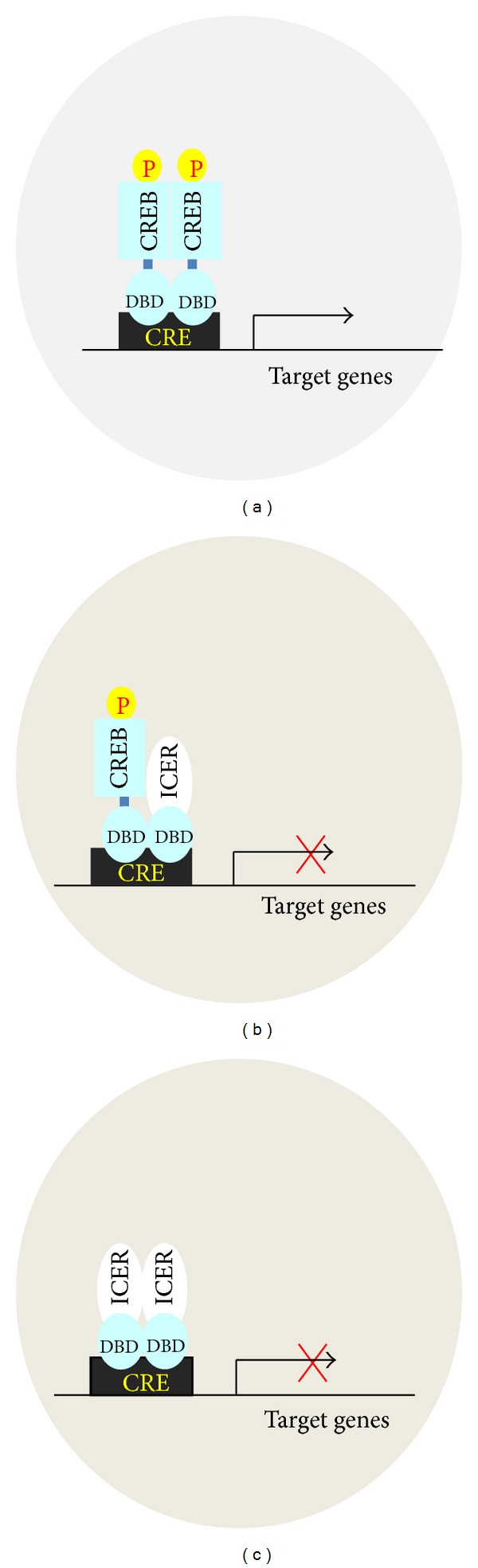
Schematic model for function of the passive repressor ICER. (a) Binding of CREB to CRE occurs when CREB is phosphorylated and the level of ICER is low. CREB can either homodimerize or form heterodimers with other activators, thereby activating gene expression. ICER competes with CREB for binding to CRE when it reaches a certain level. In this case, ICER can either (b) heterodimerize with CREB or (c) homodimerize.

ICER has been discovered as an inducible cAMP responsive element modulator (CREM) protein in neuroendocrine cells cultured with cAMP raising agents [1]. ICER is a small protein (<20 KDa), which contains one of the two CREM DNA-binding domains (DBDs) but without the activator and regulatory regions of the gene (Figure 1). CREM DBD I and DBD II are composed of a basic Leucine Zipper structure and have a strong homology with each other and with the unique DBD which is present on the CREB protein (Figure 1). Due to the presence of these two DBDs and to differential splicing, four ICER protein isoforms are possible. ICER I and ICER II isoforms contain, respectively, the DBD I and DBD II (Figure 1). These isoforms contain also the small exon *γ* of CREM gene, which instead can be missing in the two remaining isoforms: ICER I*γ* and ICER II*γ*. All four isoforms appear to be, in principle, functionally equivalent since they harbor one DBD. ICER binds cAMP response element (CRE) as homodimers and/or heterodimers with any member of the cAMP response element (CRE) binding protein/CRE modulator/activating factor 1 (CREB/CREM/ATF1) gene family [2–4]. Being composed of mainly the DBD, ICER can neither activate nor actively repress genes expression. However, when the expression level is high enough, it rather plays as a passive repressor by competing CREB/CREM/ATF1 transcriptional activators for binding to CRE (Figure 2). In mammalian cells there are thousands genes containing functional CRE [5, 6]. This implies that ICER is pivotal for regulation of genes expression in response to cAMP pathway.

ICER arises from the transcription of the *CREM* gene, directed via the P2 alternative internal intronic promoter [1]. The promoter contains a cluster of four CRE sites. Of note, ICER itself binds these sites and thereby represses its own promoter activity, in a negative feedback autoregulatory loop [7]. The kinetic of ICER induction is that of an immediate early gene, with transcript level peaking few hours after induction and thereafter rapidly declining. ICER is present in a wide array of different tissues such as nervous system, pituitary and pineal glands, thyroid, testis, liver, adipose tissue, pancreas, smooth muscles, skeletal muscle, cardiac muscle, bone, and cells of the immune system [8–17]. In the nervous system, especially in brain structures where a constitutive inhibition of cAMP-sensitive transcription seems to be necessary to maintain proper function, elevated basal level of ICER is required [18]. Notably, in the pineal gland, ICER is expressed in a circadian fashion, with high levels peaking during the subjective night followed by undetectable level in the subjective daylight [19]. This pattern of expression in the pineal gland is important for the transcriptional control of the rhythmic expression of arylalkylamine N-acetyltransferase, the rate-limiting enzyme controlling melatonin synthesis [20].

## 3. ICER as a “Brake” for Permitting Insulin Production and Secretion Return to Basal State

**Figure 3:**
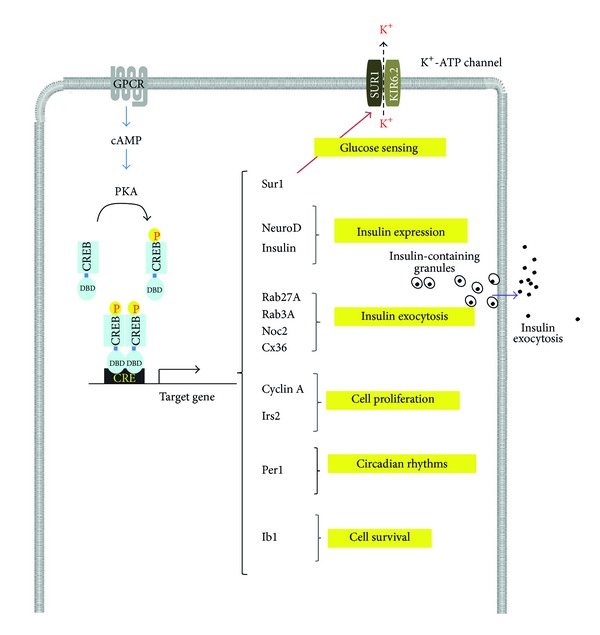
Target genes regulated by CREB and ICER in pancreatic *β*-cells. Typically phosphorylation of CREB results from the protein kinase A (PKA) activity. PKA activity is stimulated by the G protein coupled receptor-induced increase of cAMP. Some genes regulated by CREB and consequently ICER are listed in the schema. Sur1: sulfonylurea receptor 1; neurogenic differentiation 1: NeuroD; Irs2: insulin receptor 2; Per1: Period 1; Ib1: islet brain 1; Noc2: no C2 domain protein, Cx36: Connexin 36.

Insulin production, secretory function, and the rate of *β*-cells survival as well are regulated by the cAMP pathway. This is exemplified by the incretin Glucagon like peptide 1 (GLP-1), which triggers a rise of cAMP and the subsequent activation of CREB [21]. As mentioned above, thousands of genes can be regulated by CREB and ICER (some relevant targets for *β*-cells are presented on Figure 3). One of the direct targets of CREB is the neurogenic differentiation 1 transcription factor (NeuroD), which regulates the insulin expression and the sulfonylurea receptor 1 [22]. Among the other direct targets genes there are insulin itself and components of the exocytosis apparatus such as Rab3A and Rab27A, which are members of the Rab family, and two of their effectors, slp4 and Noc2 [23, 24]. The four genes contain a functional CRE able to bind ICER [25]. Overexpression of ICER in *β*-cells reduces the expression of the four secretory genes. These results have led to speculate that ICER is part of adaptive mechanism allowing the expression of the components of the secretory machinery to meet the insulin production [26]. After stimulation, insulin secretion returns to basal level. Induction of ICER could be a major mechanism permitting *β*-cells to reduce the secretory activity, while insulin expression is diminished. This *β*-cells activity is required to replenish insulin within ready releasable granules for the next meal or stimulatory conditions. Connexin 36 (Cx36) is a transmembrane protein that forms gap junctions for *β*-cells communication [27–31]. Cx36 function is required for the control of glucose-induced insulin secretion [31]. The gene coding for Cx36 contains a CRE and is negatively regulated by ICER [31], indicating that the control in the Cx36 level by ICER participates to the dynamic regulation of glucose-induced insulin secretion. Besides of regulating *β*-cell function, ICER could be instrumental for controlling *β*-cells survivals and death. In fact, *β*-cells overexpression of ICER in mice impinges *β*-cells mass by slowing proliferation [32]. Consequently, insulin secretion is collapsed and mice have developed diabetes. Direct decrease of Cyclin A expression by ICER accounts for decline in *β*-cells number in transgenic mice [33]. Insulin receptor substrate 2 (IRS-2) is required for *β*-cells proliferation and survival. IRS-2 is a target of CREB/ICER. Expression of a CREB dominant negative in *β*-cells provokes diminution of IRS-2 and activation AKT signaling, thus causing *β*-cell dysfunction and loss of *β*-cell mass [34]. The mitogen activated protein kinase (MAPK) 8 interacting protein 1 (*MAPK8IP1*) gene encodes islet brain 1 (IB1) also termed as JNK interacting protein 1, a protein that tethers kinases of the JNK pathway. The IB1 function is to preserve *β*-cells survival, insulin expression, and secretion in response to proapoptotic stimuli by regulating the c-jun N terminal kinases (JNK) pathway [16, 35]. *MAPK8IP1 *contains within its proximal regulatory region several CRE. However, only one is capable to interact with CREB and to be negatively regulated by ICER [36]. Regulation of IB1 through this sequence is crucial for the protective effect of the GLP-1 mimetic exendin-4 [37]. The protective effect of IB1 is thought to involve JNK3 activation (Figure 3).

Pancreatic *β*-cells express the molecular clock proteins controlling circadian rhythm of insulin secretion and impairment of some member of the clock genes such as circadian locomotor output cycles kaput (CLOCK) and brain and muscle ARNT-like 1 (BMAL1), leading to hypoinsulinemia and diabetes [38–41]. CLOCK and BMAL1 work through interwoven positive and negative feedback loops [42]. The two proteins form heterodimers that activate transcription of the genes coding for Period (PER) and Cryptochrome (CRY). PER/CRY heterodimers form the negative limb, which in turn inhibits the activity of CLOCK/BMAL1, thereby generating circadian rhythms of transcription/translation. At the heart of the system is the ability of CLOCK/BMAL1 heterodimers to recognize and bind the E-Box elements which are present on the promoters of both *Per* and *Cry* genes. In addition to the E-Box, *Per1* and *Per2* genes contain functional CRE elements [39, 40]. Regulation of these genes by CREB is important for the fine tuning and modulation of clock genes in response to changing environmental cues. For instance, CREB-mediated upregulation of the *Per1* gene in the suprachiasmatic nucleus neurons is required for the photic resetting that takes place during the dark-light circadian transitions [43, 44]. Moreover, CREB plays another important role in the modulation of the molecular clocks in peripheral organs, especially in the liver where it is implicated in the control of gluconeogenesis [45, 46]. Interestingly, recent data show that ICER regulates the *Per1* gene in hepatic and adrenal gland clocks [47] and such regulation could account for circadian melatonin production in the pineal gland [19]. In view of these findings, it is possible that such a mechanism could take place in *β*-cells but this remains to be addressed.

## 4. Deregulation of ICER in Response to Environmental Stressors Associated with Diabetes 

Typically, ICER activity results from a rise of its expression. Repression of target genes ensues when the expression of ICER reaches appropriate amount for competing CREB, CREM, and eventually ATF for binding to CRE (Figure 2). Such regulation could represent an adaptive mechanism for cells to return to their basal state after stimulation. In this respect, it is predictable that deregulation in the levels of ICER could strikingly perturb *β*-cells function and thereby glucose homeostasis. Several lines of evidence seem to argue in favor of such hypothesis. The first clue comes from a study carried out on Goto-Kakizaki (GK) rats, a well-characterized model of genetic nonobese type 2 diabetes in which *β*-cells function is impaired [48]. Isolated islets from these rats display high levels of CREM repressor including ICER I, indicating that the increase of ICER could contribute to *β*-cell dysfunction. Insulin secretion usually increases as the consequence of insulin resistance. However, glucose sensitivity of *β*-cells can fail to overcome insulin demand overtime. In this case overt diabetes appears. In islets of obese mice fed with a HFD, increase in the ICER level has been monitored [49]. Obesity is characterized by chronic elevation of nonesterified free fatty acids (NEFAs) including the saturated NEFA palmitate [50]. Chronic hyperglycemia resulting both from insulin resistance and glycemic excursion from the meal can also appear in obesity. There are clues that palmitate and chronic hyperglycemia may account for the increase of ICER in defective *β*-cells in obese animals. Prolonged elevation of palmitate and glucose, individually, hampers insulin secretion in human individuals and exerts harmful effects in *β*-cells. *In vitro* experiments have unveiled that increase in ICER is partly responsible of the adverse effects elicited by both diabetogenic factors.

Modification in the lipoproteins level is observed in obese individuals and is hallmark of metabolic syndrome. Increased levels of oxidized LDL-cholesterol particles together with a decrease in plasma concentration of HDL particles are seen at present as additional potential diabetogenic stressors, while they increase the risks of patients for developing cardiovascular diseases. Low plasma level of HDL and specific antibodies against oxidized LDL are found in patients with T2D. Perturbations in the two lipoproteins are further already observed in metabolic syndrome and they are worse throughout the duration of diabetes. Infusion of recombinant HDL in patients with T2D reduces glycemia by an increase in insulin secretion and glucose uptake in muscles. Improvement in insulin secretion results from cytoprotective properties of HDL by at least tackling the effects of oxidized LDL. The human modified LDL augments the expression of ICER via oxidative stress [51]. Consequently, elevation of ICER elicited by oxidized LDL cholesterol hampers insulin production and glucose-induced secretion by affecting Rab3A, Rab27A, Slp4, and Noc2. Finally cells cultured with the human oxidized LDL undergo apoptosis because of reduced expression of IB1 and JNK activity.

Transgenic mice that specifically overexpress ICER in *β*-cells exhibit high blood glucose levels throughout their lifespan and mice died from severe diabetes because of a reduced functional *β*-cell mass [32]. Chronic hyperglucagonemia usually parallels defective insulin secretion in diabetes. Glucagon acts through stimulation of the cAMP/PKA pathway, resulting in activation of CREB. As the consequence of CREB activity, the expression of ICER is induced, resulting in repression of the insulin gene transcription [52]. Induction of ICER by hyperglucagonemia may represent an additional mechanism contributing to deregulated insulin gene expression and *β*-cells failure in diabetes.

## 5. Concluding Remarks and Perspectives

While ICER represses target genes, it inhibits its own promoter as well. This negative feedback loop permits genes expression and ICER as well, returning to basal state. Elevation of ICER observed in islets *β*-cells exposed to diabetes environmental conditions raises the idea that destruction of ICER is a key for counteracting *β*-cell failure. This hypothesis is not possible if a systemic approach for silencing ICER in the body is employed. Decline of ICER is detrimental, at least for adipose function and systemic insulin sensitivity. Drastic reduction in the adipose ICER content, as observed in both obese human and mice, impairs insulin-induced glucose uptake and production of the insulin sensitizer adiponectin [16, 51]. Drop of adiponectin, if protracted in the long term, has adverse effects for systemic insulin sensitivity [53]. A careful examination in the mechanism leading to uncontrolled expression of ICER in *β*-cells needs therefore to be considered. With this regard, the rise of ICER may result either from increased activators activity or defect of the negative autoregulation. The P2 promoter activity is under the control of CREB. In *β*-cells exposed to chronic hyperglycemia the CREB level is reduced via proteasomal degradation [54]. A role for CREB in the increased production of ICER seems therefore unlikely. Future studies will be to investigate whether negative regulators are missing or rather some activators are stimulated in diabetes condition to promote sustained expression of ICER. Identification of these mechanisms would pave the way for identification of innovative therapeutic counteracting *β*-cells dysfunction and death in diabetes.
